# Cholera ante portas – The re-emergence of cholera in Kinshasa after a ten-year hiatus

**DOI:** 10.1371/currents.RRN1310

**Published:** 2012-03-12

**Authors:** Didier Bompangue, Silvan Manuel Vesenbeckh, Patrick Giraudoux, Marcia Castro, Jean-Jacques Muyembe, Benoît Kebela Ilunga, Megan Murray

**Affiliations:** ^*^Laboratoire Chrono-environnement, UMR6249, CNRS, University of Franche-Comté, Place Leclerc 25030 Besançon, France. Laboratory of Microbiology, Faculty of Medicine, University of Kinshasa, BP: 834, Kinshasa, Democratic Republic of Congo. Direction de Lutte contre la Maladie, Ministry of Public Health, Av. de la Justice 39, Gombe I, Kinshasa, Democratic Republic of Congo.; ^†^Harvard School of Public Health, Center for Communicable Disease Dynamics, 677 Huntington Avenue, Boston MA 02115, USA. Brigham and Women’s Hospital, Division of Global Health Equity, 75 Francis Street, Boston MA 02115, USA; ^‡^Laboratoire Chrono-environnement, UMR6249, CNRS, University of Franche-Comté, Place Leclerc 25030 Besançon, France; ^§^Harvard School of Public Health, Department of Global Health and Population, 677 Huntington Avenue, Boston MA 02115, USA; ^¶^Laboratory of Microbiology, Faculty of Medicine, University of Kinshasa, BP: 834, Kinshasa, Democratic Republic of Congo; ^#^Direction de Lutte contre la Maladie, Ministry of Public Health, Av. de la Justice 39, Gombe I, Kinshasa, Democratic Republic of Congo and ^**^Brigham and Women’s Hospital, Division of Global Health Equity, 75 Francis Street, Boston MA 02115, USA. Harvard School of Public Health, Department of Epidemiology, 677 Huntington Avenue, Boston MA 02115, USA.

## Abstract

Background: Cholera is an endemic disease in certain well-defined areas in the east of the Democratic Republic of Congo (DRC). The west of the country, including the mega-city Kinshasa, has been free of cases since mid 2001 when the last outbreak ended. Methods and Findings: We used routinely collected passive surveillance data to construct epidemic curves of the cholera cases and map the spatio-temporal progress of the disease during the first 47 weeks of 2011. We compared the spatial distribution of disease spread to that which occurred in the last cholera epidemic in Kinshasa between 1996 and 2001. To better understand previous determinants of cholera spread in this region, we conducted a correlation analysis to assess the impact of rainfall on weekly health zone cholera case counts between December 1998 and March 2001 and a Generalized Linear Model (GLM) regression analysis to identify factors that have been associated with the most vulnerable health zones within Kinshasa between October 1998 and June 1999. In February 2011, cholera reemerged in a region surrounding Kisangani and gradually spread westwards following the course of the Congo River to Kinshasa, home to 10 million people. Ten sampled isolates were confirmed to be Vibrio cholerae O1, biotype El Tor, serotype Inaba, resistant to trimethoprim-sulfa, furazolidone, nalidixic acid, sulfisoxaole, and streptomycin, and intermediate resistant to Chloramphenicol. An analysis of a previous outbreak in Kinshasa shows that rainfall was correlated with case counts and that health zone population densities as well as fishing and trade activities were predictors of case counts. Conclusion: Cholera is particularly difficult to tackle in the DRC. Given the duration of the rainy season and increased riverine traffic from the eastern provinces in late 2011, we expect further increases in cholera in the coming months and especially within the mega-city Kinshasa. We urge all partners involved in the response to remain alert.

Didier Bompangue and Silvan Vesenbeckh contributed equally to this work. *corresponding author: Silvan Vesenbeckh, Harvard School of Public Health (vesenbeckh@gmail.com)

Didier Bompangue is Associate Professor in the Department of Microbiology (University of Kinshasa) and

Epidemiologist in the DRC Ministry of Health. He was involved in the investigations of the described outbreak since February 2011.

**Introduction**

Cholera is a globally important disease with a devastating impact on the poor, and it is continuously advancing to previously unaffected regions of the world [1]. Since the start of the early nineties, a dramatic increase in cases has been observed globally with climate change, poverty and human mobility being among its major drivers [1] [2] [3]. The World Health Organization (WHO) estimates the burden of disease at 3-5 million cases per year [4], and in 2010, 48 countries reported disease [ [5]. Today, Haiti and sub-Saharan Africa are the most affected areas in the world; 93-98% of all cases were reported from Africa during the years 2001-2009, and in 2010, 56% of all cases were reported from Haiti[5].

The first known cases of cholera in the Democratic Republic of Congo (DRC) occurred in 1973 when cases were imported from neighboring Angola into the province Bas Congo and Kinshasa [6]. In 1978, cases were reported to have been imported from Tanzania to Kalemie, a city bordering lake Tanganyika in the east of DRC [6] [7]. Since then cholera has been reported annually from the African Great Lakes Region (AGLR) especially in a circumscribed endemic area abutting the lakes in eastern DRC [8], where a particular microenvironment seems to favor the persistence of *Vibrio cholerae [9][10][11][12][13]* *.*    

In contrast to the Lake region, the western part of the DRC including its 10 million capital Kinshasa has been only sporadically affected by cholera; there have been no primary cases reported since 2002 and only ten imported cases between 2002 and 2010. The year 2001 marked the end of a double peaked epidemic in Kinshasa (1996-1998 and 1999-2001) that lasted six years and claimed at least 5,105 cases and approximately 300 deaths (reported cases, Ministry of Health - MoH, DRC). These numbers represent documented cases during a time when cholera surveillance in DRC was being implemented and, therefore, the true burden of cholera for these years is estimated to be several times higher. Little is known about the extent and severity of this past epidemic. A recent report by the jointly EU/UN funded DRC International Humanitarian Committee dramatically underestimates its duration at three months [14].   

The WHO officially announced a new outbreak of cholera in western DRC on July 22, 2011[15] The first suspected case of cholera had been confirmed on February 23 in Lubunga, one of the five health zones of Kisangani city. Within a few weeks the disease spread further westwards following the Congo River. Despite joined efforts of the MoH, Non-Governmental Organizations (NGOs) and the United Nations (UN, notably WHO and UNICEF), the outbreak could not be contained. The aim of our study is to describe this spatio-temporal spread, and to raise awareness of a potentially devastating impact that a major cholera epidemic in the DRC and Kinshasa may have.    

## **Methods**

### Data sources

The National Integrated Disease Surveillance and Riposte System (IDSR) was established in 2000 by the MoH in conjunction with the WHO. The IDSR targets thirteen infectious diseases with epidemic potential, including cholera, for passive surveillance [16]. Suspected cases and deaths due to moderate and severe cholera are documented in each treatment facility and trained MoH officials aggregate these data at the health zone (HZ) level and report them to the MoH in Kinshasa weekly.

Re-zoning of the 306 health zones present in 2000 took place in 2003, and the number of zones increased from 306 to 515. The population per health zone in 2003 was determined through “sanitary” censuses conducted by the MoH in each newly established zone. Population estimates for each of the subsequent years up to 2011 were obtained by assuming a constant growth rate of 3% per year for all HZs.   

### Cholera case management

Following WHO policy, a suspected case of cholera is defined as ‘‘any person two years of age or older in whom acute watery diarrhea with or without vomiting develops” during a cholera epidemic. The age limit is adjusted to five years or older in interepidemic periods in order to reduce the number of false positives [17]. At the beginning of an epidemic, between five and ten samples from each HZ are laboratory confirmed through isolation of *V. cholerae* in culture. Subsequent cases of acute watery diarrhea in the same geographic region are assumed to be cholera.

Trained MoH officials with the support of NGOs ensure the field management of cholera. Patients diagnosed with cholera in local primary health care facilities are isolated on site and, whenever possible, referred to specialized treatment facilities such as Cholera Treatment Centers (CTCs) or Cholera Treatment Units (CTUs).   

### Epidemic curves and geospatial maps

Using routinely reported, weekly MoH cholera notification data, we created epidemic curves of reported cholera cases for the years 1996 to 2011, noting those periods during which data quality was known to be compromised. All maps were produced using ArcMap 10 (ESRI, Redlands, CA, USA). Shapefiles were obtained through the MoH and MONUSCO (United Nations Organization Stabilization Mission in the Democratic Republic of the Congo).   

### Cholera microbiology

The 2011 outbreak was culture confirmed by the National Cholera Laboratory in Kinshasa (Institut National de Recherche Biomédicale, INRB). From July to September 2011, 368 samples from all 7 affected DRC provinces were sent to INRB for confirmatory testing for serogroup O1; these included 149 from Kinshasa province. Ten stool samples from confirmed cases from Bandundu province, all collected on June 18, were sent to the Centers for Disease Control and Prevention (CDC, Atlanta) for further microbiological analysis including antibiotic resistance testing.   

### Rainfall and environmental variables

Daily rainfall estimates (RFE) at a 0.1° spatial resolution, generated by the National Oceanic and Atmospheric Administration’s (NOAA*)* Climate Prediction Center, were obtained through the International Research Institute for Climate and Society (IRI) for the time period 1996-2011 (http://iridl.ldeo.columbia.edu/) [18]. We report mean daily rainfall estimates for Kinshasa province and estimated the correlation between cholera case counts and rainfall during the period December 1998 – March 2001, since this was the longest period during which we found no missing data. Cross-correlation between cholera case count and rainfall was computed and expressed as a function of a time lag applied to one of the series in order to detect possible delayed effects of rainfall on the epidemic.   

To better understand the factors associated with previous spread of cholera in Kinshasa, we also examined the association between weekly case counts and geographic variables for each Kinshasa HZ from October 1998 – June 1999, the only period during that previous outbreak during which case registries were fully disaggregated by HZ. These variables represent the following health zone characteristics: area (expressed in the log scale), population, adjacency to the Congo River, presence of another major natural river, existence of a river port, occurrence of fishing and trade activity, and presence of a flood zone. The population of each HZ was obtained from the 1998 sanitary census. Adjacency to the Congo River was defined as within a 2 km buffer zone from the river; fishing and trade activity was defined as presence of a local market on the site of the port; and floodable area was defined by METTELSAT (http://www.meteo-congo-kinshasa.net/).    

We analyzed the data using GLM (Generalized Linear Model) regression models, of the negative binomial family, in order to account for the over-dispersion of cholera incidence. We used a forward selection strategy with log(population) as an offset term and included log(area), so that the HZ population density became a function of its area. We included variables in the multiple regression model using a stepwise approach that used the Akaike Index Criterion (AIC) to select the best combination of variables. Analyses were conducted using R 2.13.1, with the gamlss 4.0-8 additional package.     

## **Results**

As of November 27, 2011, 18,165 cases and 382 deaths had been reported for 2011 from 136 health zones in the DRC. This total includes 6,232 cases and 292 deaths from the four western provinces: Province Orientale, Equateur, Bandundu and Kinshasa. During the first 47 weeks of 2011, case fatality rates (CFR) in these four provinces varied from 2.58% (95% CI 1.96-3.38) in Equateur to 6.27% (95% CI 5.32-7.37) in Bandundu. The city of Kinshasa reported 663 cases and 37 deaths (CFR: 5.58%, 95% CI 4.08-7.60).   

Figure 1 shows the spread of cholera throughout 2011. Cholera cases were initially confined to the eastern provinces Katanga, South Kivu and North Kivu neighboring the lakes Tanganyika, Kivu and Edward, respectively and from there the disease gradually spread westward to zones adjacent to the Congo River. Twelve health zones surrounding Kisangani within Province Orientale were soon affected, and the first cases were reported in the province Equateur, further downstream the Congo River, during week 18. On June 13 (week 24), after only 130 days, the epidemic reached Kinshasa, around 2000 km down the river. The first case in Kinshasa province was reported in the health zone Maluku located upstream on the shore of the Congo River. This HZ is home to the biggest port at the northern gates of Kinshasa linking the city with trading cities upstream.   

**Figure:**
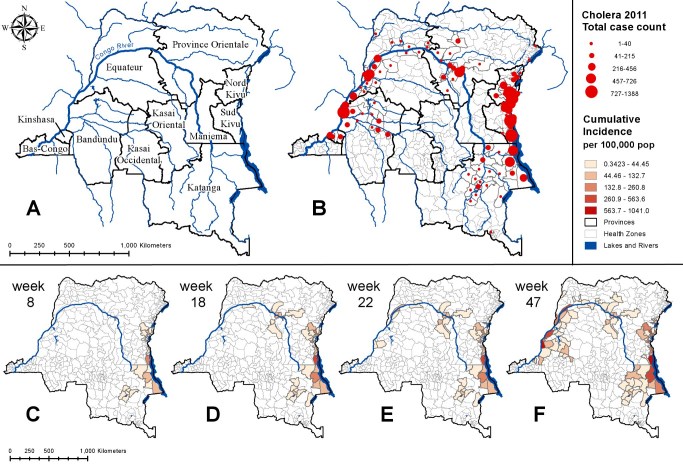
**Figure 1. Spatio-Temporal Spread of the 2011 Epidemic.** Map of the DRC with its 11 provinces and major river network (A). Total cholera case count for 493 of the health zones by week 47 (B). Cumulative incidence (per 100,000 population) at weeks 8, 18, 22 and 47 (C-F).

Figure 2 provides cholera data for Kinshasa since 1996 and shows that cholera had been absent between 2002 and 2010 and that the number of cases in the 1996-2001 outbreak rose dramatically after the onset of the rains. In the period December 1998 – March 2001 cholera cases were correlated with rainfall with a seven weeks time lag (Pearson’s r = 0.40, p < 0.001, permutation test with 1000 replications).  

**Figure:**
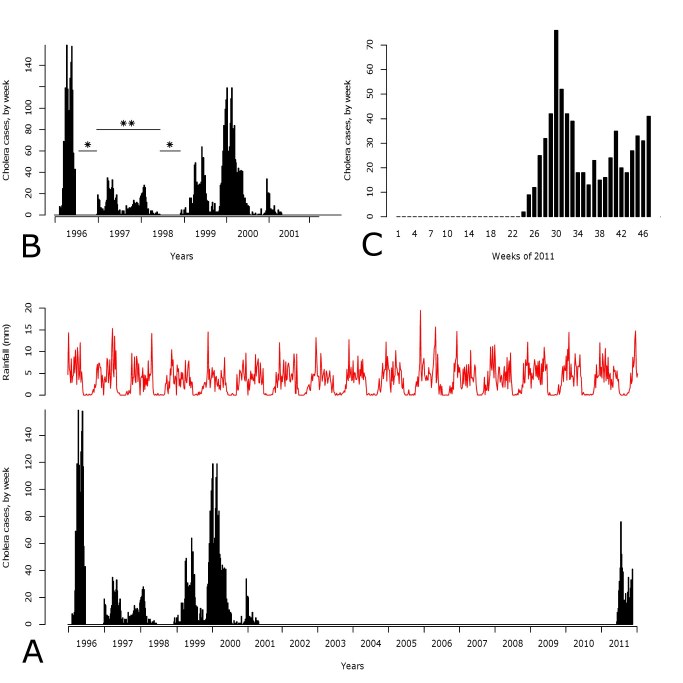
**Figure 2. Weekly Reported Cases and Estimated Rainfall 1996-2011. ** Cholera cases and estimated mean daily rainfall (in mm) by week for the period of 1996-2011 (A). Inset B highlights the 1996-2001 epidemic, indicating the periods of compromised surveillance data quality (** data available only from 1 reporting facility, * absence of data). Inset C highlights the ongoing 2011 epidemic as of week 47. 

Figure 3 compares the first affected health zones within metropolitan Kinshasa in the 1996-2001 epidemic (Barumbu, Lingwala and Kinshasa) with the 2011 epidemic (Kingabwa). Limete and Kingabwa were among the most affected health zones in both epidemics with 18% of all cases reported from them during the period 1998-1999 and more than 30% of all reported cases in 2011 originating from Kingabwa.

**Figure:**
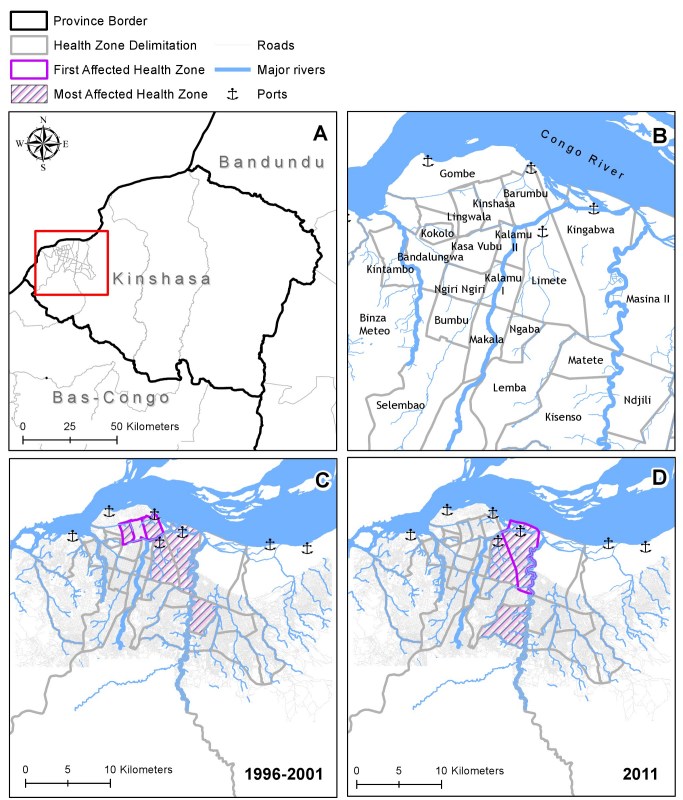
**Figure 3. Cholera in Kinshasa: Comparing the Epidemics 1996-2001 and 2011.**

The province Kinshasa and its 34 health zones, with the red box indicating metropolitan Kinshasa (A). Cholera affected areas in metropolitan Kinshasa in 1996-2001 (C) and 2011 (D). The health zone names are given in (B), and the 3 major rivers crossing the city (Bitchakutchaku, Kauka, and Ndjili from left to right) as well as the city’s major ports on the shores of Congo River are highlighted.

***Disclaimer:*** *Shapefiles were provided by the MoH and MONUSCO. The boundaries and names shown and the designations used on this map do not imply official endorsement or acceptance by the United Nations.*

Although population density, adjacency to the Congo river, the presence of a port and ongoing fishing and trade activity were found to be significant risk factors in the single factor analysis of environmental variables, Table 1 shows that population density and fishing and trade activity were the only HZ level risk factors that were retained in the multivariate model. The R^2 ^ in this final model was 77.2%.  

**Table 1**

The results of GLM regression model in the single factor (A) and the stepwise regression (B) analysis. 

**Table:** 

	A				B			
	Pr(>|t|)	Relative risk	lower limit CI95%	upper limit CI95%	Pr(>|t|)	Relative risk	lower limit CI95%	upper limit CI95%
HZ population density	0.006*	-			0.046*	-		
HZ adjacency to Congo River	0.024*	2.22	1.17	4.23	NA			
HZ presence of major river	0.097	1.66	0.94	2.93	NA			
HZ presence of port	0.02*	2.44	1.22	4.90	NA			
HZ presence of fishing and trade activity	0.005*	2.81	1.46	5.40	0.005*	2.81	1.46	5.40
HZ floodable area	0.382	1.30	0.73	2.29	NA			

Among the samples sent to INRB between July to September 2011, 15% (22/149) of those originating from Kinshasa province and 25% (91/368) of those from other provinces tested positive for *Vibrio cholerae *serogroup O1, biotype El Tor, serotype Inaba. All of the ten samples sent to CDC were confirmed to be *Vibrio cholerae *serogroup O1, biotype El Tor, serotype Inaba and shared the same antibiotic resistance pattern: resistance to trimethoprim-sulfa, furazolidone, nalidixic acid, sulfisoxaole, and streptomycin, and intermediate resistance to Chloramphenicol. All isolates were susceptible to Tetracycline and Ciprofloxacin.    

## **Discussion**

In this study, we describe the unfolding epidemic of cholera in the DRC. We find that the 2011 spatio-temporal pattern of spread is similar to that which occurred during the double peaked epidemic of 1996-2001, with cholera spreading along riverine routes from the eastern lake districts to the capital, Kinshasa. The role of the AGLR and connecting river pathways have previously been associated with cholera epidemics in neighboring Burundi [11].    

Although our descriptive analysis of surveillance data and reports is limited by some gaps in data, it nevertheless allows us to compare the areas of the city that were first affected and those that reported most of the cases during these two epidemics. We identified two predictors of Kinshasa health zone cholera burden: population density and the presence of market activity at local ports. These findings are consistent with our previous studies of determinants of cholera prevalence in endemic areas in the east of DRC [9].    

Given this pattern, we believe there is reason to fear the emergence of a major outbreak of cholera in Kinshasa. With a population of 10 million and one of the most striking growth rates worldwide (3.24% per year) [19], the expansion of the city over the past years occurred in a much faster pace than the provision of local infrastructure. Health systems are largely unprepared to manage a major outbreak of cholera in a city where 76% of its inhabitants are reported to live in slums and poor neighborhoods with very limited access to health care [20]. The water-sanitation infrastructure in the DRC is weak: the 2007 DHS data indicate a poor access to improved drinking water (48.2%) and improved sanitation (17.6%) [21], and Congolese authorities report a decline in drinking water coverage of 50% in between 1990 and 2002 [22].

The Congo River is one of the major means of transportation for the 71 million inhabitants in DRC [23]. Fishermen use rivers and lakes in eastern DRC to travel to bigger cities where they sell their fish after the end of the dry season [8]. In Kinshasa fishermen and traders are most likely to travel to the city from upstream settlements during the October to May rainy season, when waterways become more navigable. Fishermen are among the most at risk populations for cholera in DRC [8] and the influx of potentially infected people to Kinshasa may be increased during these months. In addition, our data show that heavy rains have preceded abrupt increases in caseload in the past after a delay of approximately seven weeks. These findings are consistent with several other studies from Africa, which found a positive association between rainfall and cholera case counts [8] [24] [25] [26] [27]. Reyburn et al. recently found an eight weeks time lag in a study in Tanzania [28]. It is widely accepted that climatic factors are common drivers of cholera endemic dynamics and epidemics [13] [29] [30]. Taking into account that the last epidemic occurred over ten years ago, the city’s population is likely to retain no herd immunity to the disease and to be entirely susceptible, further increasing the risk of a major epidemic.   

Cholera is particularly difficult to tackle in such a vast country as the DRC. The currently ongoing presidential elections process has the potential to further destabilize the countries weak infrastructures. An early response strategy that involves local capacity building and active participation at all levels of the local government is even more crucial. We learned from the Haiti 2010 cholera response that election processes and political instability can severely hamper the delivery of goods and services [31], and that urgently needed and already allocated funds are held back until a new government is fully functional.   

It remains to be elucidated why the epidemic could take off in western DRC in 2011 after ten years without such spread. Further research is necessary to determine factors that might have triggered this development, especially in light of a more widespread epidemic of cholera throughout Africa in 2010 and 2011. The 2011 DRC outbreak is thought to have spread to neighboring countries such as the Republic of Congo in June 2011 as well as to the Central African Republic in September 2011 [32]. Similar trends are observed in two other regions of sub-Saharan Africa: cholera is reported to have been exported from Nigeria into Cameroon and Chad, and from Ghana into Côte d'Ivoire [33].  

## **Conclusions**

The last epidemic of cholera in Kinshasa lasted for six years before it faded out in 2001. In June 2011, a new epidemic reached the city and is further spreading since then. The total case count in Kinshasa is still relatively low but there is reason to fear an upsurge of cases during the whole rainy season that lasts till the end of May 2012. Policy makers must be aware of the continued threat of cholera in Kinshasa particularly, and throughout the western DRC in general. Surveillance, prevention and treatment pose specific challenges in the presented setting and it is even more important to carefully allocate the limited resources available for this neglected tropical disease.   

## **Role of the funding source**

The project described was supported by Award Number U54GM088558 from the National Institute of General Medical Sciences. The content is solely the responsibility of the authors and does not necessarily represent the official views of the National Institute of General Medical Sciences or the National Institutes of Health. 

The funders had no role in study design, data collection and analysis, decision to publish, or preparation of the manuscript. 

## **Conflicts of interest**

The authors declare no conflicts of interest.  

## **Ethics Statement**

An ethics statement was not required for this work. 

### **Author’s contributions**

DB, BKI and JJMT collected the data, DB, MM and SV designed the study, DB, PG and SV analyzed the data, SV produced the maps, DB and SV wrote the manuscript, and MC and MM revised the manuscript. 

**Acknowledgements**

The authors thank the DRC “Cellule choléra” team for their constant participation in data collection, and Drs. Sirenda Vong as well as Xavier de Radiguès, former coordinators of Epicentre in DRC (1999 to 2003), for their help in reconstituting elements of the past epicemic in Kinshasa. We also acknowledge the contributions of the Enteric Diseases Laboratory Branch and the Waterborne Disease Prevention Branch, CDC, for laboratory testing and consultation, and thank Jeff Blossom from the Harvard Center for Geographic Analysis for technical ArcGIS support.  
